# X-Ray Supramolecular Structure, NMR Spectroscopy and Synthesis of 3-Methyl-1-phenyl-1*H*-chromeno[4,3-c]pyrazol-4-ones Formed by the Unexpected Cyclization of 3-[1-(Phenyl-hydrazono)ethyl]-chromen-2-ones

**DOI:** 10.3390/molecules16010915

**Published:** 2011-01-21

**Authors:** Itzia I. Padilla-Martinez, Irma Y. Flores-Larios, Efren V. García-Baez, Jorge Gonzalez, Alejandro Cruz, Francisco J. Martínez-Martinez

**Affiliations:** 1 Unidad Profesional Interdisciplinaria de Biotecnología del Instituto Politécnico Nacional, Av. Acueducto s/n, Barrio la Laguna Ticomán 07340, México D. F., Mexico; E-Mail: ipadillamar@ipn.mx (I.I.P.-M.); 2 Facultad de Ciencias Químicas, Universidad de Colima, km 9 Carretera Coquimatlán-Colima, Coquimatlán Colima 28400, Mexico

**Keywords:** oxidative cyclization, benzopyrano-arylhydrazone, benzopyrano-pyrazolone, pi-stacking

## Abstract

The molecular structures of nine 3-methyl-1-phenyl-1*H*-chromeno[4,3-c]pyrazol-4-one isomers, obtained by the oxidative cyclization of the corresponding 1-phenylhydrazono chromen-2-ones with copper acetate as catalyst, are reported. The molecular and supramolecular structures of the 8-chloro, 8-bromo- and 8-nitro isomers **2b-d**, were established by X-ray diffraction. The halogenated isomers **2b** and **2c** are isomorphs, they crystallize as a triclinic system, space group P-1 with two molecules in the asymmetric unit. Compound **2d** crystallizes as a monoclinic system, space group P2_1_/m with two molecules in the unit cell. The 1-phenyl ring [Cg(4)] is almost perpendicularly positioned to the chromene-pyrazole ring system. This conformation is in agreement with the anisotropic NMR shielding effect exerted by the phenyl ring over H-9 in solution. The supramolecular architecture is almost controlled by C―H···A (A = O, π) and face to face π-stacking interactions. The observed π-stacking trend between chromene and pyrazole rings is given by the overlapping between the best donor and acceptor rings in each compound.

## 1. Introduction

Pyrazole and its derivatives are shown to possess important biological and pharmaceuticalactivities [1,2] such as antimicrobial [3,4], antiviral [5,6], anxiolytic [7,8] and anti-inflammatory [4,9] activities. They are also useful in agrochemical industry as herbicides [10,11] and insecticides [12].

The 1-phenylchromeno[4,3-*c*]pyrazol-4-ones are important pyrazole derivatives which have been used for the synthesis of inmunomodulatory drugs because of their interaction with the benzodiazepine central receptor [13]. Several methods of synthesis have been reported starting from arylidenechromones and hydrazine in basic media [14,15]; 3-CN-4-[(*o*-hydroxy)phenyl]-1-phenyl-3-methylpyrazole in ethanediol [16]; 4-substituted with –OH and –Cl 1-(phenylhydrazono)-chromen-2-ones by cyclization in acidic media [17]. To the best of our knowledge, this cyclization is not expected in the absence of a 4-positioned good leaving group, and the closest reported approach is the cyclization of 6-chloro-3-{1-[(2,4,6-trichlorophenyl)-hydrazono]-ethyl}-chromen-2-one in the presence of equimolar quantities of SbCl_5_ to obtain a 3-methyl-1-(2,4,6-trichlorophenyl)-1*H*-chromeno-[4,3-*c*]pyrazol-4-one similar to **2a** in 86% yield [18] and the reaction of 1-(chloro(thiophen-2-yl)methylene)-2-phenylhydrazine with coumarin at reflux in chloroform and triethylamine to yield 1-phenyl-3-thiophen-2-yl-1*H*-chromeno[4,3-*c*]pyrazol-4-one [19]. In addition, it is worth mentioning that there are six related structures deposited in the CSD (Version of November 2008) [20] but only one discussed in the literature.

**Scheme 1:**
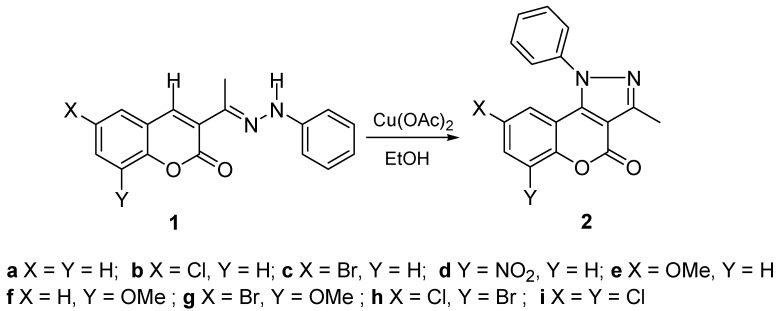
Synthesis of 3-methyl-1-phenyl-1*H*-chromeno[4,3-*c*]pyrazol-4-ones **2a-i** starting form 3-[1-(phenyl-hydrazono)-ethyl]-chromen-2-ones **1a-i**.

In this contribution the synthesis of 1-phenyl-chromeno[4,3-*c*]pyrazol-4-ones **2a-i** through the oxidative cyclization of 3-(phenyl-hydrazono)-chromen-2-ones **1a-i** with copper acetate as catalyst is reported (Scheme 1). The structures in solution by NMR as well as the molecular and supramolecular structures in the solid state, by monocrystal X-ray diffraction, are discussed.

## 2. Results and Discussion

### 2.1. Synthesis and Molecular Structure in Solution

In our efforts to crystallize hydrazone **1a** from a saturated chloroform solution, crystals of 3-methyl-1-phenyl-1*H*-chromeno[4,3-*c*]pyrazol-4-one **2a** were spontaneously formed instead in 30% yield at RT. It is worthy to note that the cyclization reaction of **1a** is not expected, because of the absence of a 4-positioned good leaving group to allow pyrazole ring formation. To ascertain the scope and limitation of this transformation, several 3-(phenyl-hydrazono)-chromen-2-ones **1b-i** were tested but cyclization did not proceed under the same conditions as for **1a**. This result lead us to use Cu(CH_3_COO)_2_·H_2_O as catalyst, since some examples of copper-catalyzed oxidative amination of alkynes [21] and azoles [22] *via* CH and NH coupling have recently been reported. Then, compounds **2a-i** were prepared in poor to good yields (50–83%), starting from the corresponding 3-[1-(phenyl-hydrazono)-ethyl]-chromen-2-ones **1a-i**, using Cu(CH_3_COO)_2_·H_2_O as catalyst in 20:1 weight ratio under mild conditions. In comparison with reported methods, starting from 4-hydroxybenzopyrano-arylhydrazones, the yields are lower or similar for **2a** (76%) [17] and **2b** (39%) [15], but in the case of **2c** (78%) and **2d** (83%) [23] they are significantly enhanced by the use of the copper catalyst.

**Scheme 2:**
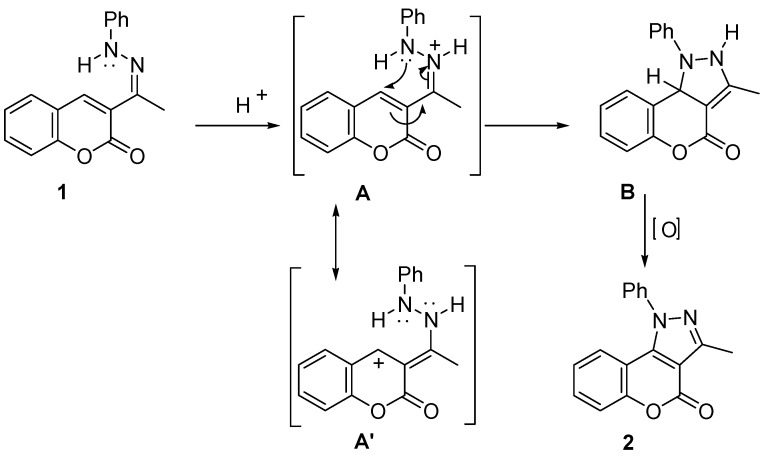
Proposed mechanism of reaction.

**Scheme 3:**
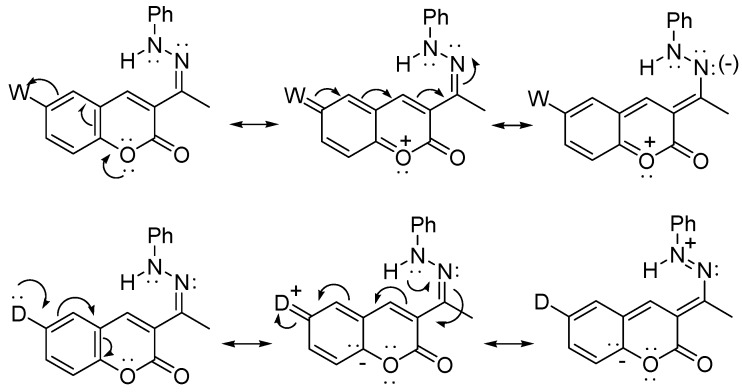
Resonance structures of 6-substituted-3-[1-(phenyl-hydrazono)-ethyl]-chromen-2-ones **1a-i** with electrowithdrawing (W) or electrodonor (D) groups.

The reaction should proceed by a simple intramolecular conjugate addition of the Ph-N to the α,β-unsaturated–C=N^+^ system, through the intermediate **A**, and the subsequent oxidation of the resulting dihydro-pyrazolone **B** (Scheme 2). This proposal is supported on similar reactions reported in acid media [24,25]. The formation of the key intermediate **A’** would be disfavored either by electro withdrawing (W) or by electrodonating (D) substituents, which would explain the necessary aid of the copper catalyst (Scheme 3).

Several differences in the ^1^H- and ^13^C-NMR spectra appear as a consequence of the cyclization. Selected NMR and IR data are listed in Table 1 and Table 2 for **1a-i** and **2a-i**, respectively. The ^1^H-NMR spectra of compounds **2a-i** is characterized by the loss of the H-4 signal, usually appearing as a singlet at δ 7.98–8.17, in the starting compounds **1a-i**. In addition, the chemical shift of H-9 in **2a-i** appears at δ 6.62–8.02, more shielded than the former H-5 (δ 6.97–8.51) in **1a-i**, because of the anisotropic NMR shielding effect exerted by the phenyl group which should be almost perpendicular to the 1-phenyl-chromeno[4,3-*c*]pyrazol-4-one ring system in compounds **2a-f**. The ^13^C chemical shift of C-3a appears at 106–107 ppm in compounds **2a-i**, whereas the former C-3, in the starting hydrazones **1a-i**, is at 127.8–130.6 ppm. Subtle shielding is also observed for C-9a (former C-10) by 7.0 ppm, in agreement with the aromatic character of the newly formed pyrazole ring. The chemical shift of C-9b (former C-4) remains almost the same even when in this position was performed the ring closure.

**Table 1:** Selected NMR and IR spectroscopic data for hydrazones **1a-i**. 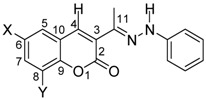

	δ ^1^H		δ ^13^C				ν/cm^−1^
Comp.	H-4	H-5	C-2	C-3	C-4	C-10	CO
**1a**	8.16	7.81	160.2	127.9	139.8	119.9	1695, 1596
**1b**	8.17	7.97	159.2	128.2	137.8	119.5	1703, 1598
**1c**	8.15	8.08	159.1	128.3	137.7	116.1	1704, 1597
**1d**	8.40	8.84	159.2	130.6	137.7	119.9	1726, 1604
**1e**	7.98	6.97	160.9	127.9	139.7	120.1	1698, 1574
**1f**	8.02	7.06	160.2	127.9	140.0	120.4	1700, 1601
**1g**	7.95	7.34	156.3	128.8	138.3	116.9	1713, 1599
**1h**	7.95	7.51	159.2	129.2	137.6	121.6	1707, 1530
**1i**	7.96	7.41	159.0	129.7	137.6	121.6	1709, 1533

**Scheme 4:**
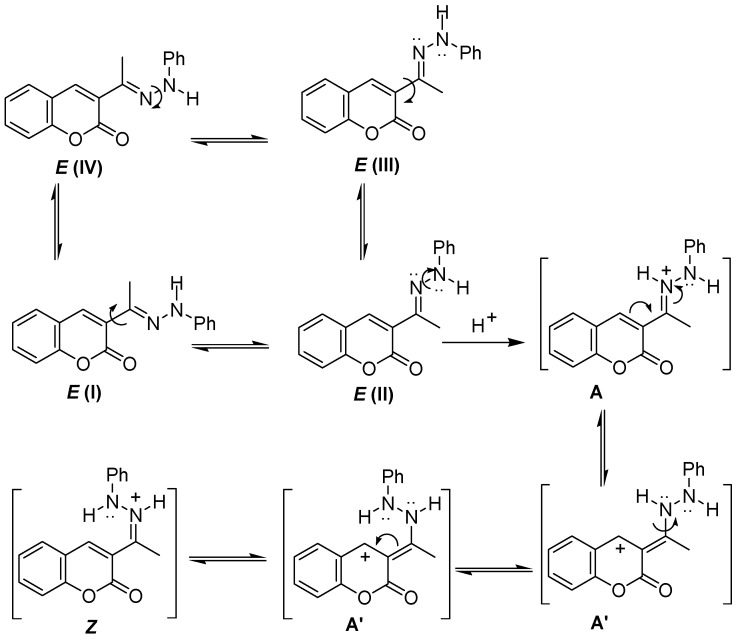
Rotamers I-IV in solution and isomerization from *E* to *Z* in acid media.

The saturation of the Me frequency in **1a** (δ 2.20, s) gives a NOE effect on proton H-4 (δ 8.16, s) and NH proton (δ 9.43, s), suggesting an *E* configuration for the C=N double bond and thus the predominance in solution of the rotamer I (Scheme 4). Thus the transformation of **1a** into **2a** implies the breaking of the double –C=N– bond to a single –C–N– to allow the location of the atoms in the proper place for cyclization in agreement with the above mentioned copper-catalyzed oxidative amination.

**Table 2:** Selected NMR and IR spectroscopic data for pyrazoles **2a-i**. 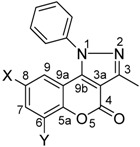

	δ ^1^H	δ ^13^C				ν/cm^−1^
Comp.	H-9	C-4	C-3a	C-9a	C-9b	CO
**2a**	7.09	158.3	106.5	112.0	141.9	1726
**2b**	7.03	157.6	106.8	113.1	140.7	1743
**2c**	7.16	157.6	106.8	113.7	140.6	1742
**2d**	8.02	156.9	106.8	112.4	143.6	1756
**2e**	6.50	158.4	106.7	112.1	141.9	1734
**2f**	6.65	157.6	106.6	112.7	142.1	1743
**2g**	6.72	156.7	106.8	113.7	140.1	1744
**2h**	6.90	156.2	106.8	112.6	140.2	1749
**2i**	6.90	156.2	106.8	114.0	140.2	1750

### 2.2. Molecular and Supramolecular Structure in Solid State

1-Phenyl-chromeno[4,3-*c*]pyrazol-4-ones **2b-d** were crystallized from saturated DMF solutions. The halogenated isomers **2b,c** crystallize as a triclinic system, space group P-1 with two molecules in the asymmetric unit. Compound **2d** crystallizes as a monoclinic system, space group P2_1_/m with two molecules in the unit cell. A summary of bond lengths and angles are listed in Table 3 and crystal data and structure refinement for **2b-d** are listed in Table 4. As in other coumarin derivatives, the replacement of Cl by Br does not alter the crystal packing [26]. All the atoms of pyrazole and chromenone rings lie in a single plane within the limits of experimental error. The 1-phenyl ring in compounds **2b-d** is sterically hindered and appears twisted by 71.9(2)º, 74.7(5)º and 92.1(2)º, respectively, from the three ring fused coplanar chromeno[4,3-*c*]pyrazol-4-one system in agreement with the conformation observed in solution (*vide supra*). The torsion angle between both planes is very close to that observed for 1-phenyl-1*H*-chromeno[4,3-*c*]pyrazol-4-one of 73.1(6)º [27]. However, in compound **2d** the 1-phenyl ring [*Cg(4)*] is almost perpendicularly positioned, thus a symmetry plane cut the molecule through its equatorial plane and only one half of the phenyl ring is observed. This conformation is in agreement with the observed anisotropic NMR shielding effect exerted by the phenyl ring over H-9 in solution.

**Figure 1:**
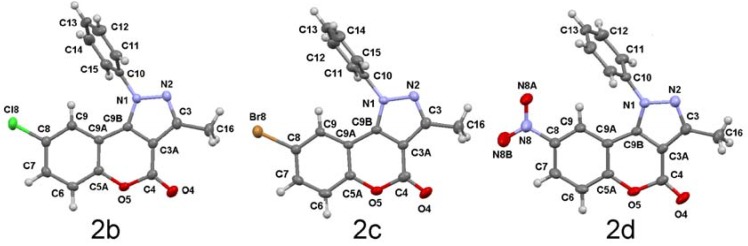
The molecular structures of **2b-d**, from left to right, showing the atom-labelling scheme. Displacement ellipsoids are drawn at the 30% probability level and H atoms are shown as small spheres of arbitrary radii.

The molecular structures of the three isomers are very similar and the major differences among them arise from the nature of the 8-substituent, Figure 1. A brief comparison with the starting coumarins points out the lengthening of C9a―C9b bond length to 1.439(5) Å (mean value of **2b-d**), from a mean reported value of 1.35 Å (C3―C4 in the former coumarins) [28], in agreement with a delocalized electronic character of the pyrazole ring. 

**Figure 2:**
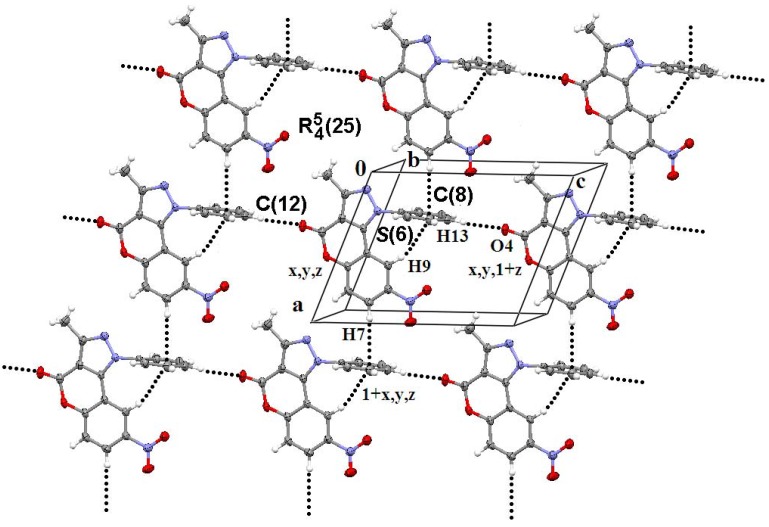
Supramolecular structure of compound **2d** in the *ac* plane. *S(6)* intramolecular ring and C(8) chain forming bifacial C―H···π interactions, *C(12)* chain and *R^5^_4_(25)* ring motifs are also observed.

Because of the arrangement of the aromatic rings, the supramolecular architecture is almost controlled by C―H···A (A = O, π) and face to face π-stacking interactions, whose geometrical parameters are listed in Table 4. In the solid state C9―H9···*Cg(4)* and C9···*Cg(4)* distances, and C9―H9···*Cg(4)* angle, suggest an intramolecular C―H···π interaction *S(6)* in **2d**, Figure 2. Even when these geometric parameters are similar among **2a-d**, only those corresponding to **2d** lie are in the proper range to be considered as such [29].

**Table 3:** Selected bond lengths and angles from X-ray data of compounds **2b-d**. 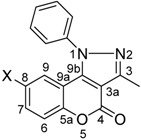

	2b X = Cl	2c X = Br	2d X = NO2
Atoms	Bond lengths (Å)		
X(8)―C(8)	1.732(2)	1.894(4)	1.466(2)
O(4)―C(4)	1.200(2)	1.197(6)	1.189(2)
O(5)―C(4)	1.385(2)	1.385(6)	1.404(2)
O(5)―C(5A)	1.382(2)	1.379(5)	1.374(2)
N(1)―N(2)	1.376(2)	1.374(5)	1.379(2)
N(1)―C(9B)	1.346(2)	1.353(5)	1.345(2)
N(1)―C(10)	1.433(2)	1.428(6)	1.433(2)
N(2)―C(3)	1.321(2)	1.315(6)	1.315(2)
C(3)―C(3A)	1.408(3)	1.400(7)	1.408(3)
C(3A)―C(4)	1.435(3)	1.441(6)	1.441(3)
C(3A)―C(9B)	1.384(2)	1.378(5)	1.380(2)
C(5A)―C(9A)	1.402(3)	1.389(6)	1.403(3)
C(9A)―C(9B)	1.437(3)	1.438(5)	1.442(2)
O(8B)―N(8)			1.195(3)
O(8A)―N(8)			1.204(2)
	**Bond angles (º)**		
C(4)―O(5)―C(5A)	123.60(15)	123.8(4)	124.12(15)
N(2)―N(1)―C(9B)	111.82(14)	111.3(3)	111.53(12)
N(2)―N(1)―C(10)	118.91(15)	119.8(4)	121.02(14)
C(9B)―N(1)―C(10)	129.18(15)	128.9(3)	127.44(14)
N(1)―N(2)―C(3)	105.86(15)	105.9(4)	105.81(15)
C(3)―C(3A)―C(4)	131.53(15)	131.9(4)	132.13(14)
C(3)―C(3A)―C(9B)	106.46(16)	106.5(4)	106.24(15)
O(4)―C(4)―O(5)	116.68(17)	117.0(4)	115.98(18)
O(5)―C(4)―C(3A)	114.98(14)	114.5(4)	114.44(14)
X(8)―C(8)―C(7)	119.13(15)	119.0(4)	119.30(18)
N(1)―C(9B)―C(3A)	105.87(15)	105.9(3)	106.10(15)

**Table 4:** Geometric parameters associated with D―H···A (A = O, π) interactions for compounds **2a–d**.

Comp.	D―H···A^a^ (symmetry code)	H···A/Å	D···A/Å	D―H···A/º
**2a^b^**	C6―H6···*Cg(4)* (x, y, 1 + z)	2.89	3.820(3)	178
	C9―H9···*Cg(4)* (x, y, z)	2.99	3.825(3)	150(2)
	C16―H16A···*Cg(3)* (−x, −½ + y, −z)	2.75(3)	3.6659(18)	157
**2b**	C13―H13···O4 (x, y, z − 1)	2.400	3.265(7)	155
	C15―H15···O5 (2 − x, 1 − y,1 − z)	2.570	3.443(6)	157
	C7―H7···*Cg(4)* (x, y − 1, z)	2.57	3.460(2)	161
	C16―H16C···*Cg(3)* (1 − x, 1 − y, −z)	2.78	3.535(2)	136
**2c**	C13―H13···O4 (x, y, z + 1)	2.450	3.340(7)	161
	C15―H15···O5 (−x, 1 − y, −z)	2.580	3.450(6)	156
	C7―H7···*Cg(4)* (x, 1 + y, z)	2.72	3.631(5)	167
	C16―H16B···*Cg(3)* (1 − x, 1 − y, −z)	2.87	3.633(5)	137
**2d**	C13―H13···O4 (x, y, z + 1)	2.53	3.464(3)	179
	C7―H7···*Cg(4)* (1 + x, y, z)	2.78	3.6999(3)	171
	C9―H9···*Cg(4)*	2.79	3.632(3)	152

^a^ *Cg(3)* the centroid of the benzenoid ring (C5AC9AC9C8C7C6C5A) and *Cg(4)* the centroid of the phenyl ring (C10―C15); ^b^ From reference 32.

**Figure 3:**
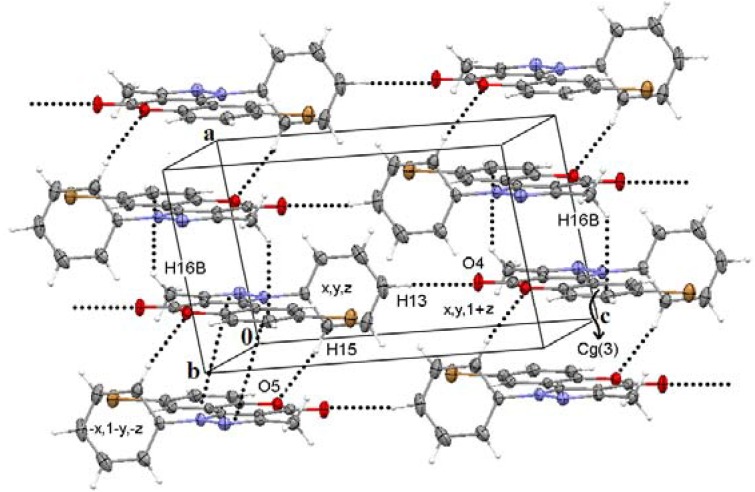
Intermolecular interactions for molecule **2c** in the *ac* plane. *C(12)* chain motif is observed through the participation of C15―H15···O5 and C16―H16B···*Cg(3)* contacts running along the direction of the *a* axis.

The first dimension (1-D) is directed by C13—H13···O4C4 interactions, between an aromatic hydrogen and the oxygen of the lactone group, developing *C(10)* chains along the direction of the *c* axis in **2b-d**. Molecules of **2b,c** self assemble in the *bc* plane and **2d** in the *ac* plane through C7―H7···*Cg(4)* interactions forming *C(8)* chains. The 2-D assembly is thus described as a *R^5^_4_(25)* ring, in agreement with the graph set notation conventions [30], Figure 2. 2-D assembled monolayers of **2b,c** and **2d** are face-to-face π-stacked developing the 3-D along the *a* and the *b* axis, respectively. A *C(12)* chain motif complements the 3-D in compounds **2b,c** through the participation of C15―H15···O5 and C16―H16B···*Cg(3)* contacts running along the direction of the *a* axis (Figure 3).

The participation of the N-phenyl ring [*Cg(4)*] in π-stacking is restricted to C―H···π interactions because of its disposition out of the plane. In contrast, the remaining pyrazole [*Cg(1)*], pyrone [*Cg(2)*] and benzenoid [*Cg(3)*] rings are lying in the same plane and thus are appropriately positioned for π-stacking. The geometric parameters associated with π-stacking interactions are listed in Table 5. Pyrazole ring is stacked with pyrone ring in compound **2a** [*Cg(1)*···*Cg(2)*] [31], it further appears stacked with the Cl- or Br- substituted benzenoid ring [*Cg(1)*···*Cg(3)*] in compounds **2b** and **2c**. In both compounds, the π-stacking between pyrone and benzenoid rings, typical of coumarins, is also observed [*Cg(2)*···*Cg(3)*]. However, in the case of compound **2d** only *Cg(1)* and *Cg(3)* are stacked, the EW group 8-NO_2_ diminishes the charge transfer capability of the benzenoid ring, enabling the formation of π-stacked centrosymmetric pairs with pirazole ring, the best charge transfer donor ring. In the other hand, the donor-acceptor capabilities of the benzenoid ring changes on going from **2a** to **2d**, according with the increase of the EW nature of the 8-substituent. Thus, the observed π-stacking trend between the rings is given by the overlapping between the best donor and acceptor ring in each molecule. This trend is consistent with those observed for other CCDC deposited structures [32], whose molecular and supramolecular analysis is missing (LOLZER, LOLZOB, LOLZUH, LOMBAQ, LOMBEU). Compounds **2a-2d** are functional isomers but only **2b** and **2c** are isomorphous, however the supramolecular structure of all of them is almost the same, varying only in the π-stacked rings and the propagating directions of the supramolecular motifs.

**Table 5:** Geometric parameters associated with π···π stacking interactions for compounds **2a–2d**.

Comp.	Centroids^a^ (symmetry code)	Intercentroid distance/A°	Dihedral angle/º	Interplanar distance/A°
**2a^b^**	*Cg(1)*···*Cg(2)* (−x, −½ + y, −z)	3.8508(9)	0.000	3.4916(1)
**2b**	*Cg(1)* · *Cg(2)* (1 − x, 1 − y, 1 − z)	3.6117(10)	0.30(8)	3.3563(7)
	*Cg(1)*···*Cg(3)* (2 − x, 1 − y, 1 − z)	3.6664(11)	1.33(9)	3.3697(7)
	*Cg(2)*···*Cg(3)* (2 − x, 1 − y, 1 − z)	3.6345(11)	1.23(8)	3.4103(6)
**2c**	*Cg(1)*···*Cg(2)* (1 − x, 1 − y, 1 − z)	3.708(2)	0.3(2)	3.4328(17)
	*Cg(1)*···*Cg(3)* (−x, 1 − y, −z)	3.727(2)	1.0(2)	3.4367(17)
	*Cg(2)*···*Cg(3)* (−x, 1 − y, −z)	3.6345(11)	1.23(8)	3.4103(6)
**2d**	*Cg(1)*···*Cg(3)* (1 − x, −½ + y, −z)	3.8523(8)	0.02(8)	3.5032(7)

^a^* Cg(1)* is the centroid of the pyrazole ring (N1N2C3C3AC9B), *Cg(2)* the centroid of the pyrone ring (O5C4C3AC9BC9AC5A), *Cg(3)* the centroid of the benzenoid ring (C5AC9AC9C8C7C6C5A) and *Cg(4)* the centroid of the phenyl ring (C10-C15); ^b^ From reference 32 (LOLZUH).

It is noteworthy that in these compounds, neither –Cl, –Br or –NO_2_ substituents in the benzenoid ring nor the lactone carbonyl, are involved in dipole-dipole interactions [33,34]. This observation contrasts with most of the coumarins studied by our group, whose supramolecular architectures are strongly influenced by the participation of these groups in multicentered interactions [35,36].

## 3. Experimental

### 3.1. Materials and Methods

All chemicals and solvents were of reagent grade and used as received. The starting coumarins were synthesized as reported elsewhere [33]. Melting points were measured on an Electrothermal IA 9100 apparatus and were uncorrected. IR spectra were recorded neat using a Varian 3100 FT-IR EXCALIBUR series spectrophotometer. ^1^H- and ^13^C-NMR spectra were recorded on a Varian Mercury 300 (^1^H, 300.08; ^13^C, 75.46 MHz) instrument in CDCl_3_ solutions, unless otherwise is specified, chemical shifts are in ppm and coupling constants in Hz, measured with SiMe_4_ as internal reference. Mass spectra were obtained in a GC-MS system (Saturn 2100T) with an electron ionization mode (Hewlett-Packard 5972 series) using HP5. Elemental analyses were performed on a Perkin-Elmer 2400 elemental analyzer.

### 3.2. X-ray Data Collection and Structure Determination

Crystals suitable for X-ray analysis were obtained by slow crystallization from saturated DMF solutions. Single-crystal X-ray diffraction data for molecules **2b-d** were collected on a Bruker Apex II area detector diffractometer at 293 K with Mo Kα radiation, λ = 0.71073 Å. A semiempirical absorption correction was applied using SADABS [37], and the program SAINT [37] was used for integration of the diffraction profiles. The structures were solved by direct methods using SHELXS97 [38] program of WinGX package [39]. The final refinement was performed by full-matrix least-squares methods on *F*^2^ with SHELXL97 program [37]. H atoms on C, N and O were positioned geometrically and treated as riding atoms, with C―H = 0.93–0.98 Å, and with *U*iso(H) = 1.2*U*eq(C). Mercury was used for visualization, molecular graphics and analysis of crystal structures [40], software used to prepare material for publication was PLATON [41]. Crystallographic data (excluding structure factors) for the structures in this paper have been deposited with the Cambridge Crystallographic Data Centre as supplementary publication CCDC numbers 766071 **2b**, 766070 **2c**,766072 **2d**. Crystal data and details concerning data collection and structure refinement are given in Table 6.

**Table 6:** Crystal data and structure refinement details for **2b-d**.

	2b	2c	2d
Chemical formula	C_17_H_11_N_2_O_2_Cl_1_	C_17_H_11_N_2_O_2_Br_1_	C_17_H_11_N_3_O_4_
Mw	310.7	355.19	321.2
Cell setting, Space group	Triclinic, P-1	Triclinic, P-1	Monoclinic, P 2_1_/m
a (A°)	7.1177 (8)	7.1681(8)	9.4294(11)
b (A°)	9.2540 (10)	9.3210(11)	7.0064(8)
c (A°)	11.7266(13)	11.8449(14)	12.0294(14)
α (º)	110.450(2)	109.820(2)	90
β (º)	98.468(2)	97.016(2)	112.826(2)
γ (º)	97.748(2)	96.891(2)	90
V (Å ^3^)	701.14(8)	727.83(15)	732.50(7)
Z	2	2	2
Density (mg cm^−3^)	1.471	1.621	1.46
μ (mm^−1^)	0.281	2.831	0.11
Crystal form, color	Block, pale yellow	Block, colorless	Block, pale yellow
Crystal size (mm^3^)	0.48 × 0.22 × 0.19	0.40 × 0.20 × 0.20	0.45 × 0.33 × 0.30
No. of measured,	6092	7652	4922
independent and	3160	2853	2514
observed reflections	2840	2013	2261
R_int_	0.024	0.054	0.024
*θ*_max_(°)	28.3	26	28.3
Refinement on	F^2^	F^2^	F^2^
R[F^2^ > 2*σ*(F^2^)], *w*R(F^2^),S	0.048, 0.116, 1.089	0.057, 0.116, 1.029	0.043, 0.122, 1.056
No. of reflections	3160	2853	2514
No. of parameters	200	200	218
Weighting scheme	1/[σ^2^(Fo^2^) + (0.0542P)^2 ^+ 0.2899P]	1/[σ^2^(Fo^2^) + (0.0542P)^2 ^+ 0.1266P]	1/[σ^2^(Fo^2^) + (0.0576P)^2 ^+ 0.321P]
	P = (Fo^2^ + 2Fc^2^)/3	P = (Fo^2^ + 2Fc^2^)/3	P = (Fo^2^ + 2Fc^2^)/3
Δ*ρ*_max_, Δ*ρ*_min_ (eÅ^−3^)	0.411, −0.281	0.670, −0.322	0.194, −0.199

### 3.3. General Methods of Synthesis

6-Substituted-3-[1-(phenylhydrazono)-ethyl]-chromen-2-ones **1a-i**. were synthesized from phenyl-hydrazine and 0.5 g of the corresponding coumarins, following standard procedures. The syntheses of compounds **2a** [15,17], **2b** [15], **2c**, **2d** [23] have been reported elsewhere, albeit with lack of some spectroscopic data, thus for completeness purposes they are included but elemental analysis was performed only to the new compounds **2e-f**.

*3-[1-(Phenylhydrazono)-ethyl]-chromen-2-one* (**1a**). Obtained from 3-acetyl-2*H*-1-benzopyran-2-one (0.5 g, 2.66 mmol) and phenylhydrazine (0.26 mL, 2.66 mmol) as an orange solid in 85% yield (0.633 g, 2.26 mmol), mp = 193–196 °C, IR ν_neat_ (cm^−1^): 3295 (N-H), 1695 (OC=O), 1596 (C=O), 1255, 1155 (C-O). ^1^H-NMR (DMSO-d6) δ: 9.43 (s, 1H, NH), 8.16 (s, 1H, H-4), 7.81 (d, 1H, H-5,^ 3^*J* = 7.7), 7.57 (dd, 1H, H-7, ^3^*J* = 8.0, 7.5), 7.38 (d, 1H, H-8, ^3^*J* = 8.3, ^3^*J* = 8.3), 7.33 (t, 1H, H-6,^ 3^*J* = 8.0, 7.6,^4^*J* = 2.2), 6.74–7.24 (m, 5H, Ph), 2.20 (s, 3H, CH_3_). ^13^C-NMR (DMSO-d6) δ: 160.2 (C-2), 153.6 (C-9), 146.2 (C-11), 139.8 (C-4), 139.2 (C*i*), 132.2 (C-7), 129.5 (C-5), 129.5 (C*m*), 127.9 (C-3), 125.2 (C-6), 120.0 (C*p*), 116.4 (C-8), 119.9 (C-10), 113.7 (C*o*), 15.8 (Me). Anal. Calcd for C_17_H_14_N_2_O_2_; C, 73.37; H, 5.07; N, 10.12. Found: C, 73.27; H, 4.91; N, 10.12. *m/z* = 277.1 (M, 22%), 77 (20%).

*6-Chloro-3-[1-(phenylhydrazono)-ethyl]-chromen-2-one* (**1b**). Obtained from 3-acetyl-6-cloro-2*H*-1-benzopyran-2-one (0.5 g, 2.22 mmol) and phenylhydrazine (0.22 mL, 2.22 mmol) as an orange solid in 82% yield (0.578 g, 1.83 mmol), mp = 184–188 °C, IR ν_neat_ (cm^−1^): 3300 (N-H), 1703 (OC=O), 1598 (C=O), 1251, 1155 (C-O), 810 (C-Cl). ^1^H-NMR (DMSO-d6) δ: 9.49 (s, 1H, NH), 8.17 (s, 1H, H-4), 7.97 (d, 1H, H-5, ^4^*J* = 2.3), 7.57 (dd, 1H, H-7, ^3^*J* = 8.8, ^4^*J* = 2.3), 7.43 (d, 1H, H-8, ^3^*J* = 8.8), 6.75–7.25 (m, 5H, Ph), 2.20 (s, 3H, CH_3_). ^13^C-NMR (DMSO-d6) δ: 159.2 (C-2), 151.6 (C-9), 145.4 (C-11), 138.1 (C*i*), 137.8 (C-4), 130.9 (C-7), 129.0 (C-6), 128.8 (C*m*), 128.2 (C-3), 127.7 (C-5), 120.8 (C*p*), 119.5 (C-10), 117.8 (C-8), 113.1 (C*o*), 15.0 (Me). *m/z* = 312 (M, 30%), 313 (8%), 240 (8%), 77 (28%).

*6-Bromo-3-[1-(phenylhydrazono)-ethyl]-chromen-2-one* (**1c**). Obtained from 3-acetyl-6-bromo-2*H*-1-benzopyran-2-one (0.5 g, 1.87 mmol) and phenylhydrazine (0.18 mL, 1.87 mmol) as an orange solid in 67% yield, (0.451 g, 1.25 mmol), mp = 184–186 °C, IR ν_neat_ (cm^−1^): 3301 (N-H), 1704 (OC=O), 1597 (C=O), 1250, 1158 (C-O), 681 (C-Br). ^1^H-NMR (DMSO-d6) δ: 9.48 (s, 1H, NH), 8.15 (s, 1H, H-4), 8.08 (d, 1H, H-5, ^4^*J* = 2.3), 7.68 (dd, 1H, H-7, ^3^*J* = 8.8, ^4^*J* = 2.3), 7.35 (d, 1H, H-8,^3^*J* = 8.8), 6.75–7.23 (m, 5H, Ph), 2.17 (s, 3H, CH_3_). ^13^C-NMR (DMSO-d6) δ: 159.1 (C-2), 152.0 (C-9), 145.4 (C-11), 138.1 (C*i*), 137.7 (C-4), 133.8 (C-7), 130.8 (C-5), 128.9 (C*m*), 128.3 (C-3), 121.3 (C-6), 119.5 (C*p*), 118.1 (C-8), 116.1 (C-10), 113.1 (C*o*), 15.0 (Me). *m/z* = 356 (M, 100%), 358 (30%), 357 (20%), 278 (5%), 77 (27%).

*6-Nitro-3-[1-(phenylhydrazono)-ethyl]-chromen-2-one* (**1d**)**.** Obtained from 3-acetyl-6-nitro-2*H*-1-benzopyran-2-one (0.5 g, 2.14 mmol) and phenylhydrazine (0.21 mL, 2.14 mmol) as an orange solid in 53% yield (0.370 g, 1.14 mmol), mp = 204–206 °C, IR ν_neat_ (cm^−1^): 3328 (N-H), 1726 (OC=O), 1604 (C=O), 1516, 1340 (C-NO_2_), 1239, 1113 (C-O). ^1^H-NMR (DMSO-d6) δ: 9.55 (s, 1H, NH), 8.84 (d, 1H, H-5, ^4^*J* = 2.6), 8.35 (dd, 1H, H-7, ^3^*J* = 9.1, ^4^*J* = 2.6), 8.40 (s, 1H, H-4), 7.60 (d, 1H, H-8, ^3^*J* = 9.1), 6.76-7.78 (5H, -Ph), 2.20 (s, 3H, CH_3_). ^13^C-NMR (DMSO-d6) δ: 159.2 (C-2), 157.1 (C-9), 144.4 (C-11), 144.2 (C-6), 137.7 (C-4), 137.5 (C*i*), 130.6 (C-3), 129.6 (C*m*), 126.1 (C-7), 124.2 (C-5), 121.5 (C*p*), 119.9 (C-10), 117.7 (C-8), 113.6 (C*o*), 13.7 (Me). *m/z* = 322 (M, 20%), 246 (5%), 77 (15%).

*6-Methoxy-3-[1-(phenylhydrazono)-ethyl]-chromen-2-one* (**1e**). Obtained from 3-acetyl-6-methoxy-2*H*-1-benzopyran-2-one (0.5 g, 2.29 mmol) and phenylhydrazine (0.23 mL, 2.29 mmol) as an orange solid in 72% yield (0.512 g, 1.65 mmol), mp = 147–149 °C, IR ν_neat_ (cm^−1^): 3303 (N-H), 1698 (OC=O), 1574 (C=O), 1243, 1134 (C-O). ^1^H-NMR δ: 7.98 (s, 1H, H-4), 7.63 (s, 1H, NH), 7.24 (d, 1H, H-8, ^3^*J* = 8.1), 7.05 (dd, 1H, H-7, ^3^*J* = 9.1, ^4^*J* = 2.1), 6.86–7.30 (m, 5H,-Ph), 6.97 (d, 1H, H-5,^4^*J* = 2.4), 2.28 (s, 3H, CH_3_). ^13^C-NMR δ: 160.9 (C-2), 156.3 (C-6), 148.4 (C-9), 144.8 (C-11), 139.7 (C-4), 139.3 (C*i*), 129.5 (C*m*), 127.9 (C-3), 120.9 (C*p*), 120.1 (C-10), 119.7 (C-7), 117.6 (C-8), 110.2 (C-5), 113.5 (C*o*), 14.1 (Me). *m/z* = 307 (M, 24%), 230 (5%), 77 (15%).

*8-Methoxy-3-[1-(phenylhydrazono)-ethyl]-chromen-2-one* (**1f**). Obtained from 3-acetyl-8-methoxy-2*H*-1-benzopyran-2-one (0.5 g, 2.29 mmol) and phenylhydrazine (0.23 mL, 2.29 mmol) as an orange solid in 91% yield (0.647 g, 2.09 mmol), mp = 152–156 °C, IR ν_neat_ (cm^−1^): 3306 (N-H), 1700 (OC=O), 1601 (C=O), 1263, 1160 (C-O). ^1^H-NMR δ: 8.02 (s, 1H, H-4), 7.59 (s, 1H, NH), 7.28 (d, 1H, H-7, ^3^*J* = 7.7), 7.17 (t, 1H, H-6, ^3^*J* = 7.7), 7.06 (d, 1H, H-5, ^3^*J* = 7.7), 6.87-7.36 (m, 5H, Ph), 2.29 (s, 3H, CH_3_). ^ 13^C-NMR δ: 160.2 (C-2), 147.1 (C-8), 144.7 (C-11), 140.0 (C-4), 143.5 (C-9), 139.3 (C*i*), 129.5 (C*m*), 127.9 (C-3), 124.6 (C-5), 120.9 (C-6), 120.4 (C-10), 120.0 (C*p*), 113.5 (C*o*), 113.4 (C-7), 14.1 (Me). *m/z* = 306.2 (M, 100%), 230 (5%), 77 (17%).

*6-Bromo-8-methoxy-3-[1-(phenylhydrazono)-ethyl]-chromen-2-one* (**1g**). Obtained from 3-acetyl-6-bromo-8-methoxy-2*H*-1-benzopyran-2-one (0.5 g, 1.68 mmol) and phenylhydrazine (0.16 mL, 1.68 mmol) as an orange solid in 74% (0.485 g, 1.25 mmol), mp = 185–188 °C, IR ν_neat_ (cm^−1^) 3312 (N-H), 1713 (OC=O), 1599 (C=N), 1258 (C-O). ^1^H-NMR δ: 7.95 (s, 1H, H-4), 7.58 (s, 1H, NH), 7.34 (s, 1H, H-5), 7.24 (s, 1H, H-7), 6.84–7.29 (m, 5H, Ph), 2.28 (s, 3H, CH_3_), 3.96 (s, 3H, OMe). ^13^C-NMR δ: 156.3 (C-2), 151.6 (C-8), 147.8 (C-9), 144.5 (C-11), 138.5 (C-13), 138.3 (C-4), 129.5 (C*m*), 128.8 (C-3), 121.1 (C*p*), 122.0 (C-5), 121.4 (C-6), 116.9 (C-10), 116.5 (C-7), 113.5 (C*o*), 56.7 (MeO–), 13.8 (Me). *m/z* = 386 (M, 100%), 308 (5%), 77 (20%).

*8-Bromo-6-chloro-3-[1-(phenylhydrazono)-ethyl]-chromen-2-one* (**1h**). Obtained from 3-acetyl-8-bromo-6-chloro-2*H*-1-benzopyran-2-one (0.5 g, 1.66 mmol) and phenylhydrazine (0.16 mL, 1.66 mmol) as an orange solid in 84% yield (0.548 g, 1.39 mmol), mp = 199–201 °C, IR ν_neat_ (cm^−1^) 3312 (N-H), 1707 (OC=O), 1530 (C=N), 1248 (C-O). ^1^H-NMR δ: 7.95 (s, 1H, H-4), 7.62 (s, 1H, NH), 7.71 (d, 1H, H-7, ^4^*J* = 2.4), 7.51 (d, 1H, H-5, ^4^*J* = 2.4), 6.90-7.29 (m, 5H, Ph.), 2.29 (s, 3H, CH_3_). ^13^C-NMR δ:159.2 (C-2), 144.3 (C-11), 140.2 (C-9), 137.8.5 (C-13), 137.6 (C-4), 134.2 (C-7), 130.1 (C-6), 129.6 (C-14), 129.2 (C-3), 126.9 (C-5), 121.6 (C-10), 113.5 (C-15), 110.7 (C-8), 13.8 (Me). *m/z* = 390.1 (M, 100%), 391.1 (30%), 392.0 (25%), 315 (5%), 76.9 (30%).

*6,8-Dichloro-3-[1-(phenylhydrazono)-ethyl]-chromen-2-one* (**1i**). Obtained from 3-acetyl-6,8-dichloro-2*H*-1-benzopyran-2-one (0.5 g, 1.95 mmol) and phenylhydrazine (0.19 mL, 1.95 mmol) as an orange solid in 82% yield (0.557 mg, 1.60 mmol), mp = 196–198 °C, IR ν_neat_ (cm^−1^) 3311 (N-H), 1709 (OC=O), 1533 (C=N), 1162 (C-O). ^1^H-NMR δ: 7.96 (s, 1H, H-4), 7.62 (s, 1H, NH), 7.55 (d, 1H, H-7,^4^*J* = 2.2), 7.41 (2, 1H, H-5, ^4^*J* = 2.2), 6.94–7.30 (m, 5H, Ph), 2.29 (s, 3H, CH_3_). ^13^C-NMR δ: 159.0 (C-2), 151.3 (C-9), 144.3 (C-11), 138.8 (C-13), 137.6 (C-4), 134.2 (C-6), 131.3 (C-7), 129.8 (C*m*), 129.7 (C-3), 124.2 (C-8), 121.6 (C-10), 121.3 (C*p*), 113.6 (C*o*), 13.8 (Me). *m/z* = 347 (M, 20%), 346.3 (55%), 274 (8%), 77 (30%).

*3-Methyl-1-phenyl-1H-chromeno[4,3-c]pyrazol-4-one* (**2a**). Cu(CH_3_COO)_2_·H_2_O (0.025 g, 0.125 mmol) was dissolved in ethyl alcohol (20 mL) and added to a solution of **1a** (0.500 g, 1.78 mmol) and ethyl alcohol (30 mL). The mixture was refluxed during 3 h, the resulting solid was filtered, washed with cold ethyl alcohol (5 mL) and several times with distilled water, air dried and recrystallized from ethyl acetate to obtain 0.372 mg (1.34 mmol) of **2a** as a white powder in 76% yield, mp = 227–230 °C, IR ν_neat_ (cm^−1^): 1726 (OC=O), 1272, 1202 (C-O). ^1^H-NMR δ: 7.44 (t, 1H, H-7, ^3^*J* = 8.6, ^4^*J* = 1.6 Hz), 7.40 (d, 1H, H-6, ^3^*J* = 8.1), 7.09 (d, 1H, H-9, ^3^*J* = 7.9), 7.02 (t, 1H, H-8, ^3^*J* = 7.9, ^4^*J* = 1.6), 7.52–7.62 (m, 5H, Ph), 2.67 (s, 3H, CH_3_). ^13^C-NMR δ: 158.3 (C-4), 153.4 (C-5a), 151.0 (C-3), 141.9 (C-9b), 139.5 (C*i*), 131.3 (C-7), 130.4 (C*p*), 130.1 (C*o*), 127.0 (C*m*), 124.1 (C-8), 122.6 (C-9), 118.2 (C-6), 112.0 (C-9a), 106.5 (C-3a), 13.1 (Me). *m/z* = 276.2 (M, 100%), 247.3 (5%), 206.2 (14%), 77.0 (16%).

*8-Chloro-3-methyl-1-phenyl-1H-chromeno[4,3-c]pyrazol-4-one* (**2b**). Obtained as described for **2a** starting from **1b** (0.500 g, 1.59 mmol) to give **2b** (0.343 g, 1.10 mmol, 69% yield) as a pale yellow powder, mp = 280–283 °C, IR ν_neat_ (cm^−1^): 1743 (OC=O), 1204 (C-O), 814 (C-Cl). ^1^H-NMR δ: 7.58 (dd, 1H, H-7 ^3^*J* = 8.8, ^4^*J* = 1.9), 7.35 (d, 1H, H-6 ^3^*J* = 8.8), 7.03 (d, 1H, H-9 ^4^*J* = 1.9), 7.38–7.65 (m, 5H, Ph), 2.68 (s, 3H, CH_3_). ^13^C-NMR δ: 157.6 (C-4), 151.8 (C-5a), 151.2 (C-3), 140.7 (C-9b), 139.0 (C*i*), 131.2 (C-7), 130.8 (C-*p*), 130.2 (C-*o*), 129.5 (C-8), 126.9 (C-*m*), 122.3 (C-9), 119.6 (C-6), 113.1 (C-9a), 106.8 (C-3a), 13.1 (Me). *m/z* = 310.2 (M, 100%), 311.0 (70%), 309.3 (45%), 275.3 (5%), 77 (22%).

*8-Bromo-3-methyl-1-phenyl-1H-chromeno[4,3-c]pyrazol-4-one* (**2c**). Obtained as described for **2a** starting from **1c** (0.500 g, 1.39 mmol) to afford **2c** (0.388 g, 1.09 mmol, 78% yield) as a white powder, mp = 278–280 °C, IR ν_neat_ (cm^−1^): 1742 (OC=O), 1266, 1203 (C-O). ^1^H-NMR δ: 7.52 (dd, 1H, H-7), 7.28 (d, 1H, H-6, ^3^*J* = 8.9), 7.16 (d, 1H, H-9 ^4^*J* = 2.4), 7.54–7.78 (m, 5H, Ph), 2.67 (s, 3H, CH_3_). ^13^C-NMR δ: 157.6 (C-4), 152.3 (C-5a), 151.2 (C-3), 140.6 (C-9b), 139.0 (C*i*), 134.0 (C-7), 130.8 (C*p*), 130.3 (C*o*), 126.9 (C*m*), 125.3 (C-9), 119.9 (C-8), 116.8 (C-6), 113.7 (C-9a), 106.8 (C-3a), 13.1 (Me). *m/z* = 354.3 (M, 80%), 356.1 (100%), 356.9 (35%), 358.0 (5%), 274.3 (5%), 77 (25).

*3-Methyl-8-nitro-1-phenyl-1H-chromeno[4,3-c]pyrazol-4-one* (**2d**). Obtained as described for **2a** starting from **1d** (0.500 g, 1.54 mmol) to give **2d **(0.412 g, 1.28 mmol, 83% yield) as a pale yellow powder, mp = 248–254 °C, IR ν_neat_ (cm^−1^): 1756 (OC=O), 1259, 1207 (C-O), 1519 (C-NO_2_). ^1^H-NMR δ: 8.31 (dd, 1H, H-7, ^3^*J* = 9.1, ^4^*J* = 2.6), 8.02 (d, 1H, H-9 ^4^*J* = 2.6), 7.55 (d, 1H, H-6, ^3^*J* = 9.1), 7.56–7.72 (m, 5H, Ph), 2.71 (s, 3H, CH_3_). ^13^C-NMR δ: 156.9 (C-4), 156.6 (C-5a), 151.5 (C-3), 143.6 (C-9b), 140.2 (C-8), 138.6 (C*i*), 131.2 (C*p*), 130.6 (C*o*), 126.7 (C*m*), 126.0 (C-7), 119.3 (C-9), 118.8 (C-6), 112.4 (C-9a), 106.8 (C-3a), 13.1 (Me). *m/z* = 321.0 (M, 100%), 320.2 (25%), 322.9 (5%), 275.3 (10%), 77 (21%).

*8-Methoxy-3-methyl-1-phenyl-1H-chromeno[4,3-c]pyrazol-4-one* (**2e**). Obtained as described for **2a** starting from **1e** (0.500 g, 1.61 mmol) to obtain **2e** (0.258 g, 0.84 mmol, 52% yield) as a white powder, mp = 232–234 °C, IR ν_neat_ (cm^−1^): 1734 (OC=O), 1238, 1203 (C-O). ^1^H-NMR δ: 7.32 (d, 1H, H-6,^3^*J* = 9.0), 6.98 (dd, 1H, H-7, ^3^*J* = 9.0, ^4^*J* = 3.1), 6.50 (d, 1H, H-9, ^4^*J* = 3.1), 7.63-7.54 (m, 5H, Ph), 2.68 (s, 3H, CH_3_). ^13^C-NMR δ: 158.4 (C-4), 155.6 (C-8), 151.0 (C-3), 147.9 (C-5a), 141.9 (C-9b), 139.5 (C*i*), 130.5 (C*o*), 130.5 (C*p*), 127.4 (C*m*), 119.2 (C-7), 118.8 (C-6), 112.1 (C-9a), 106.7 (C-3a), 105.5 (C-9), 13.4 (Me). *m/z* = 306.1 (M, 100%), 291.3 (28%), 277 (3%), 77 (22%). Anal. Calcd. for C_18_H_14_N_2_O_3_; C, 70.58; H, 4.61; N, 9.14. Found: C, 70.22; H, 4.50; N, 9.00. 

*6-Methoxy-3-methyl-1-phenyl-1H-chromeno[4,3-c]pyrazol-4-one* (**2f**). Obtained as described for **2a** starting from **1f** (0.500 g, 1.61 mmol) to give **2f** (0.248 g, 0.806 mmol, 50% yield) as a white powder, mp = 238–240 °C, IR ν_neat_ (cm^−1^): 1743 (OC=O), 1273, 1207 (C-O). ^1^H-NMR δ: 7.02 (dd, 1H, H-7,^3^*J* = 8.2, 7.6), 6.97 (t, 1H, H-8, ^3^*J* = 7.6, 8.2), 6.65 (dd, 1H, H-9 ^3^*J* = 7.6, ^4^*J* = 1.5), 7.54–7.62 (m, 5H, Ph), 2.69 (s, 3H, CH_3_). ^13^C-NMR δ: 157.6 (C-4), 151.0 (C-3), 148.4 (C-6), 143.3 (C-5a), 142.1 (C-9b), 139.6 (C*i*), 130.4 (C*p*), 130.0 (C*o*), 127.2 (C*m*), 123.9 (C-8), 114.1 (C-9), 112.9 (C-7), 112.7 (C-9a), 106.6 (C-3a), 13.2 (Me). *m/z* = 306.1 (M, 100%), 291.3 (5%), 277 (20%), 77 (22%). Anal. Calcd. for C_18_H_14_N_2_O_3_; C, 70.58; H, 4.61; N, 9.14. Found: C, 70.83; H, 4.70; N, 9.00.

*8-Bromo-3-methyl-6-methoxy-1H-chromeno[4,3-c]pyrazol-4-one* (**2g**). Obtained as described for **2a** starting from **1g** (0.500 g, 1.28 mmol) to obtain **2g** (0.393 g, 1.01 mmol, 79% yield) as a pale yellow powder, mp = 289–292 °C, IR ν_neat_ (cm^−1^): 1744 (OC=O), 1275, 1205 (C-O). ^1^H-NMR δ: 6.72 (s, 1H, H-9), 7.06 (s, 1H, H-6), 7.51-7.62 (m, 5H, Ph), 2.67 (s, 3H, CH_3_). ^13^C-NMR δ: 156.7 (C-4), 151.1 (C-5a), 148.9 (C-3), 142.3 (C-6), 140.1 (C-9b), 139.0 (C-10), 130.7 (C-9), 130.1 (C-11), 127.0 (C-12), 123.9 (C-5), 123.3 (C-8), 116.5 (C-13), 116.1 (C-7), 113.7 (C-9a), 106.8 (C-3a), 13.1 (Me). *m/z* = 384.5 (M, 80%), 386.2 (100%), 385.5 (25%), 357.5 (10%), 290.5 (10%), 77.0 (25%). Anal. Calcd. for C_18_H_13_N_2_O_3_Br; C, 56.13; H, 3.40; N, 7.27. Found: C, 55.88; H, 3.40; N, 7.20.

*6-Bromo-8-Chloro-3-methyl-1H-chromeno[4,3-c]pyrazol-4-one* (**2h**). Obtained as described for **2a** starting from **1h** (0.5 g, 1.27 mmol) to obtain **2h** (0.249 g, 0.64 mmol, 50% yield) as a pale yellow powder, mp = 259–261 °C, IR ν_neat_ (cm^−1^): 1749 (OC=O), 1277, 1224 (C-O). ^1^H-NMR δ: 6.90 (s, 1H, H-9), 7.82 (s, 1H, H-7), 7.53-7.65 (m, 5H, Ph), 2.65 (s, 3H, CH_3_). ^13^C-NMR δ: 156.2 (C-4), 151.1 (C-5a), 148.2 (C-3), 140.2 (C-9b), 138.8 (C-10), 134.2 (C-7), 130.9 (C-9), 130.6 (C-11), 129.5 (C-8), 126.9 (C-12), 121.4 (C-13), 113.9 (C-6), 112.6 (C-9a), 106.8 (C-3a), 13.0 (Me). *m/z* = 390.0 (M, 100%), 389.5 (60%), 388.5 (62%), 310 (5%), 77(25%). Anal. Calcd. for C_17_H_10_N_2_O_2_BrCl; C, 52.40; H, 2.59; N, 7.19. Found: C, 52.70; H, 2.63; N, 7.00.

*6,8-Dichloro-3-methyl-1H-chromeno[4,3-c]pyrazol-4-one* (**2i**). Obtained as described for **2a** starting from **1i** (0.5 g, 1.43 mmol) to obtain **2i** (0.259 g, 0.74 mmol, 52% yield) as a pale yellow powder, mp = 224–226 °C, IR ν_neat_ (cm^−1^): 1750 (OC=O), 1225 (C-O). ^1^H-NMR δ: 6.90 (s, 1H, H-9), 7.47 (s, 1H, H-7), 7.52-7.64 (m, 5H, Ph), 2.65 (s, 3H, CH_3_). ^13^C-NMR δ: 156.2 (C-4), 151.1 (C-5a), 147.8 (C-3), 140.2 (C-9b), 138.8 (C-10), 131.3 (C-7), 130.9 (C-9), 130.3 (C-11), 129.1 (C-8), 126.9 (C-12), 124.1 (C-6), 120.8 (C-13), 114.0 (C-9a), 106.8 (C-3a), 13.0 (Me). *m/z* = 344.5 (M, 100%), 346.2 (80%), 345.3 (68%), 308.5, 77 (22%). Anal. Calcd. for C_17_H_10_N_2_O_2_Cl_2_; C, 59.15; H, 2.92; N, 8.11. Found: C, 58.90; H, 2.89; N, 8.00.

## 4. Conclusions

3-Methyl-1-phenyl-1*H*-chromeno[4,3-*c*]pyrazol-4-one (**2a**) spontaneously crystallizes from CHCl_3_solutions of 3-[1-(phenyl-hydrazono)-ethyl]-chromen-2-one (**1a**) whereas the 6-substituted isomers **1b-i** failed to do so, requiring Cu(CH_3_COO)_2_·H_2_O as catalyst to yield the corresponding 1-phenyl-chromeno[4,3-*c*]pyrazol-4-ones **2b-i** in moderate to good yields (50–83%) under mild conditions. The NMR data in solution and the X-ray data in the solid state are consistent with the *N*-phenyl ring almost perpendicular to the three fused rings chromeno-pyrazole system. In the solid state this geometrical arrangement of the aromatic rings determines the supramolecular architecture by C―H···A (A = O, π) and face to face π-stacking interactions which are very similar among **2b-d**, varying only in the nature of the π-stacked rings and in the propagating direction. The observed π-stacking trend between chromeno and pyrazole rings is given by the overlapping between the best donor and acceptor rings in each molecule, modulated by the electronic character of the X and Y substituents.
